# Inter -and intraobserver variation of ultrasonographic cartilage thickness assessments in small and large joints in healthy children

**DOI:** 10.1186/1546-0096-7-12

**Published:** 2009-06-04

**Authors:** Anne Helene Spannow, Mogens Pfeiffer-Jensen, Niels Trolle Andersen, Elisabeth Stenbøg, Troels Herlin

**Affiliations:** 1 Department of Pediatrics, Aarhus University Hospital, Skejby, Aarhus, Denmark; 2 Department of Rheumatology, Aarhus University Hospital Norrebrogade, Aarhus, Denmark; 3 Department of Biostatistics, Institute of Public Health, Aarhus University, Aarhus, Denmark

## Abstract

**Background:**

There is an increasing interest among pediatric rheumatologist for using ultrasonography (US) in the daily clinical examination of children with juvenile idiopathic arthritis (JIA). Loss of joint cartilage may be an early feature of destructive disease in JIA. However, US still needs validation before it can be used as a diagnostic bedside tool in a pediatric setting. This study aims to assess the inter- and intraobserver reliability of US measurements of cartilage thickness in the joints of healthy children.

**Methods:**

740 joints of 74 healthy Caucasian children (27 girls/47 boys), aged 11.3 (7.11 – 16) years were examined with bilateral US in 5 preselected joints to assess the interobserver variability. In 17 of these children (6 girls/11 boys), aged 10.1(7.11–11.1) years, 170 joints was examined in an intraobserver sub study, with a 2 week interval between the first and second examination.

**Results:**

In this study we found a good inter- and intraobserver agreement expressed as a coefficient of variation (CV) less than 10% in the knee (CV = 9.5%_interobserver _and 5.9%_intraobservserI_, 9.3%_intraobserverII _respectively for the two intraobserver measurements) and fairly good for the MCP joints (CV = 11.9%_interobserver_, 12.9%_intraobserverI _and 11.9%_intraobsevrerII_). In the ankle and PIP joints the inter- and intraobserver agreement was within an acceptable limit (CV<20%) but not for the wrist joint (CV>26%). We found no difference in cartilage thickness between the left and right extremity in the investigated joints.

**Conclusion:**

We found a good inter -and intraobserver agreement when measuring cartilage thickness with US. The inter- and intraobserver variation seemed not to be related to joint size. These findings suggest that positioning of the joint and the transducer is of major importance for reproducible US measurements. We found no difference in joint cartilage thickness between the left and right extremity in any of the examined joint of the healthy children. This is an important finding giving the opportunity of using the non-affected extremity as a reference when assessing articular joint cartilage damage in JIA.

## Background

There is an increasing interest among pediatric rheumatologist for using musculoskeletal ultrasonography as an investigative tool for children with juvenile idiopathic arthritis (JIA) [1-3]. In JIA, early detection of inflammatory joint pathologies ideally would allow clinicians to initiate relevant therapies in a timely manner preventing destruction of the cartilaginous tissue and periarticular bone and subsequently improve morbidity and long-term outcome. Accumulating evidence in adult RA patients suggest that musculoskeletal ultrasonography (US) is superior in the detection and monitoring of early inflammatory and destructive joint changes [4,5]. In patients with early, untreated oligoarthritis US revealed a high prevalence of subclinical disease[5].

US give the opportunity for precise and detailed pathoanatomical images. It has a number of practical advantages compared to other imaging techniques, such as magnetic resonance imaging (MRI), including being non-invasive, easily repeatable, safe, and relatively inexpensive. It allows dynamic real-time assessment in many joints from different anatomical regions. Ultrasonography requires no sedation, is easy to perform bedside and children seems to accept the examination.

However, there has been some concern regarding its reproducibility and operator dependability. While systematic studies on different aspects of validation of US in RA are now emerging [6-11]documented validity assessment has only been described in few studies in pediatric patients[2,12,13].

Loss of joint cartilage may be an early feature of destructive disease in JIA and when using high frequency US the cartilage, appearing anechoic, is easily visualized [3,14-16]. In a pilot study, we found good agreement of interobserver variability in assessments of joint cartilage thickness in a small group of healthy children[13]. Recently, we described a good level of agreement between MRI and US measurements of hyaline cartilage in healthy children [12].

The present study was undertaken to assess inter- and intraobserver reliability of US assessments of cartilage thickness in small and large joints from clinically dominant joint regions in a large group of healthy children. In addition we wanted to evaluate whether recommended EULAR standard scans was applicable in a pediatric setting.

## Methods

### Subjects

Ten joints from each of 74 healthy Caucasian children (27 girls/47 boys), aged 11.3 (7.11 – 16) years were examined by two investigators. The unequal distribution between boys and girls is due to the fact that more boys than girls accepted to participate. All children were examined with bilateral US in 5 preselected joints (right and left knee, ankle, wrist, metacarpophalangeal (MCP) and proximal interphalangeal (PIP)) to assess the interobserver variability in measurements of joint cartilage thickness. All examinations were carried out within a period of two months. In seventeen of the 74 children (6 girls/11 boys), aged 10.1 (7.11–11.1) years, the 10 joints described above, were re-examined within a 2 week interval to access the intraobserver variation.

All children were free from known chronic diseases including musculoskeletal diseases. They had no history of joint trauma, swelling, tenderness or previous surgical interventions in the joints. A joint examination prior to the US examination was performed to confirm the clinical normality of the children's joints. None of the children was taking medicine influencing on growth or bone metabolism including corticosteroids. No sport activities were allowed on the examination day prior to the US investigation. The parents of all participants gave informed consent. The study was conducted in accordance with the Helsinki II declaration, and was approved by the local ethical committee of Aarhus, Denmark.

### Investigators

All US examinations (inter – and intraobserver study) were carried out by two observers (AHS (observer I) and MP (observer II) with experience in musculoskeletal ultrasonography. Prior to the study the two observers reached consensus on defining the bony landmarks for measurements of cartilage thickness in the five examined joints. The sonographers carried out the US examinations immediately after each other. US images were stored on DVD for later entry in a database. The US measurements of cartilage thickness from each child were performed blinded to the observers.

### Ultrasonography

For the ultrasonographic examination, we used conventional B-mode obtained on a real-time Hitachi EUB-6500 CFM scanner, equipped with a linear 6–13 MHz transducer. Scanner settings were uniform for all measurements. The US image acquisition time was approximately 20 – 30 minutes for each child. The EULAR standard US scan planes were used[12,17].

### Knee and ankle joints

**Figure 1:**
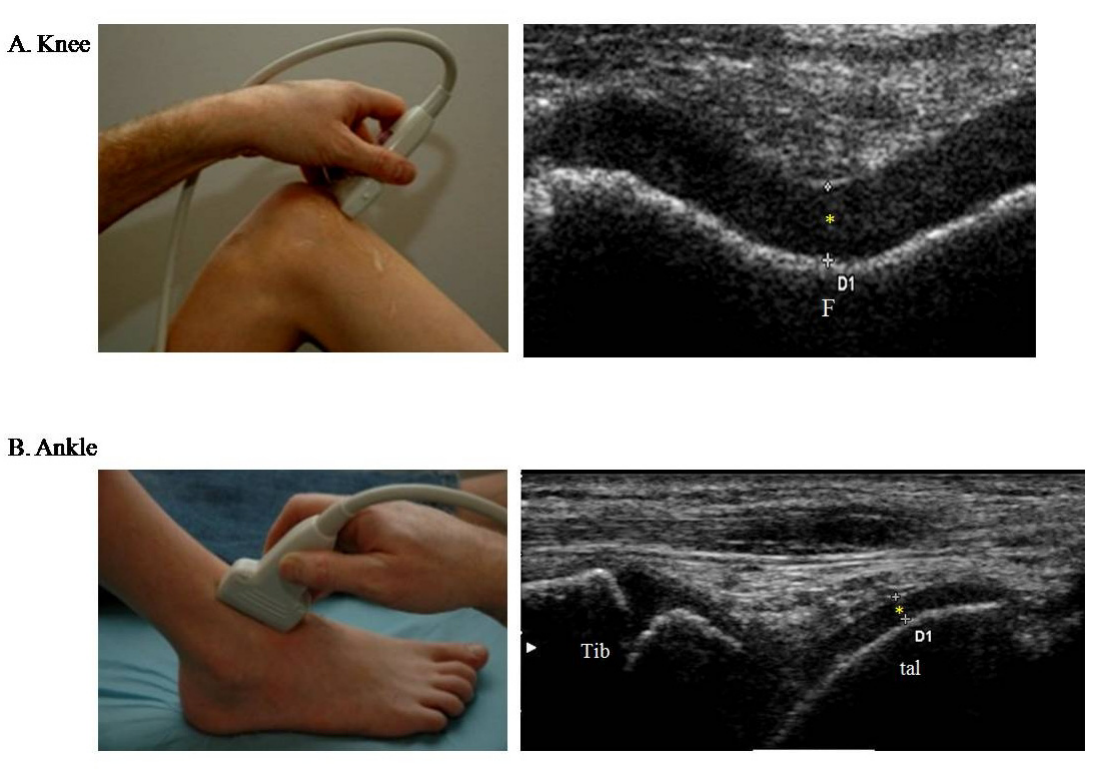
**US ultrasonographic standard scans for cartilage thickness measurements in the knee and ankle joints**. * = hyaline cartilage, F = femur, Tib = tibia, tal = talus.

For cartilage thickness measurements of the knee, the child was placed in a supine position and a suprapatellar transverse scan with the knee in maximal flexion was performed according to EULAR guidelines[17]. Cartilage thickness was measured corresponding to the midline of the intercondylar notch (Fig. 1A).

The ankle joint was examined with the plantar surface of the foot resting on the examination bed (knee in 90-degree flexion) and an anterior longitudinal scan between the first and second metatarsal bone was obtained [17]. The anterior demarcation of the cartilage on the medial part of the dome of talus was identified. From this point, a distance of 5 mm in proximal direction was measured out and the cartilage thickness was measured perpendicular to the bone surface (Fig. 1B).

### Wrist and finger joints

Cartilage thickness measurements of the wrist were obtained with the child in supine position, with both hands palm-side down on the examination bed and placed aside the body.

**Figure 2:**
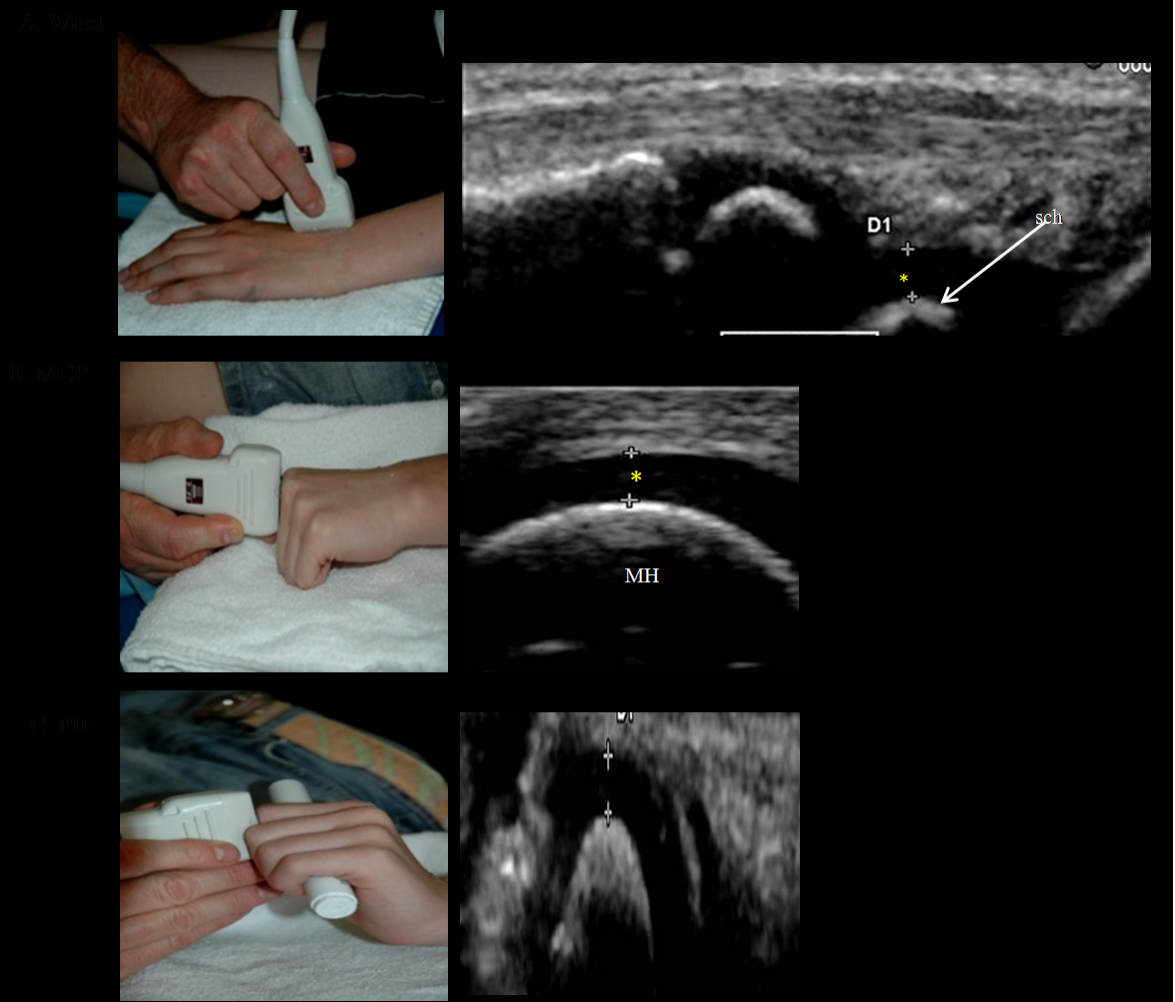
**Ultrasonographic standard scans for cartilage thickness measurements in the wrist, metacarpophalangeal (MCP) and proximal interphalangeal (PIP)**. * = hyaline cartilage, R = radius, sch = os schaphoideum, MH = metacarpal head, DP = distal phalange.

Wrist cartilage thickness measurements were obtained with a dorsal longitudinal scan corresponding to the articulating surface of the radial and scaphoid bones [17] (Fig. 2A).

With the child in the same position as mentioned for wrist measurements cartilage thickness of the second MCP and second PIP joints was obtained from a longitudinal dorsal scan with the MCP and PIP joints in 90-degree flexion[17] (Fig. 2B &2C).

### Statistical analysis

Analysis of the interobserver agreement of cartilage thickness measurements consists of a systematic variation (whether one observer has a tendency to measure thicker or thinner cartilage thickness) and a random variation (e.g. biological variation within joints in the child). The systematic variation can be corrected if known, whereas this is not possible for the random variation. The systematic interobserver variation was described in absolute terms calculated from differences (mm) and compared by paired t-test. Significant level was set to 5% (*p *< 0.05) in all calculations.

**Figure 3:**
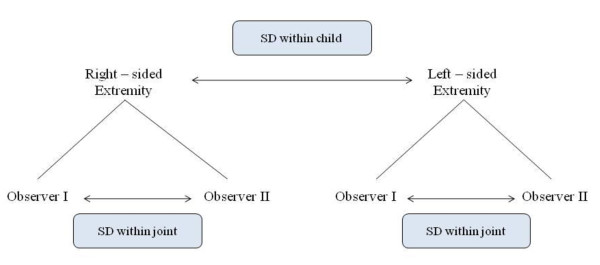
**Shows the definition of Random Variation, standard deviation "within child" and "within joints", for the inter and intraobserver cartilage thickness measurements in the examined children**. SD = standard deviation.

The random variation between the two observer (random interobserver variation) was given by the standard deviation (SD) in mm and was divided into two parts: one common for the two right- and left-sided joints on the same child (SD^2^_within child_) and one corresponding to the additional variation for each joint (SD^2^_within joint_). See Figure 3.

The total variation between the two observers SD^2^_total _= SD^2^_within child _+ SD^2^_within joint _is calculated. The variation between the two observers is the combination of both intraobserver variation and the additional interobserver variation.

With regard of intraobserver agreement, the statistical analysis was divided into a systematic intraobserver variation within an observers repeated measurements using the same US standard scans and equipment on two separate examination days, two weeks apart.

The random intraobserver variation can be described in the same terms as in the section of interobserver random variation and were expressed as the SD in mm and was divided into two parts: one part common for two equivalent joints of the same child (SD^2^_within child_) and the other part the additional variation of each joint (SD^2^_within joint_).

The standard deviations (SD) calculated from the relative differences of measurements were estimates of the coefficient of variation (CV = SD/mean × 100) and expressed the level of agreement in both inter- and intraobserver study. We considered a coefficient of variation ≤ 10% for good and ≤ 20% as acceptable [18]. In all the analyses, systematic and random variations between the cartilage thickness on the right and on the left side were included.

The Proc Mixed procedure in the statistical package SAS 9.1 was used for the analyses. Paired t-test was used for comparing the systematic intraobserver variation. Significant level was set to 5% (*p *< 0.05) in all calculations.

## Results

We found a significant systematic interobserver variation for the knee, ankle, MCP and PIP joint, but not for the wrist (p = 0.172, Additional file 1). These findings express that one of the observers had a tendency to systematically measure a thicker cartilage thickness compared to the other observer, even though the observers had made detailed discussions on consensus for the anatomical landmarks of cartilage thickness measurements. The random interobserver variation was largest in the standard deviation for the "within joint" variable.

As an expression for the level of agreement between observers (total variation) the coefficient of variation was estimated as described in the statistical section. A good level of agreement between the two observers was found in the knee and MCP joints with a CV of 9.5% and 11.9% respectively. The coefficient of variation for the ankle and PIP joints where within an acceptable limit (<20%) but the CV for the wrist joint (26%) was found to be poor.

With regard to the intraobserver variation there seems to be a good reproducibility when using US for cartilage thickness measurements (Additional files 2&3).

We found no difference in the analyses of the systematic and random variation between the cartilage thickness on the left and right side of the examined joints see Additional file 4.

Because our results in the study of inter- and intraobserver variation showed a tendency towards a systematic inter- and intraobserver variation, we conducted re-measurements of cartilage thickness on the stored US images. Consensuses for pointing out cartilage borders on the stored images were reassured by the observers (AHS and MP). The US images (knee, ankle, wrist, and MCP and PIP joints) obtained from 19 randomly selected children out of the 74 previously examined children in the interobserver study and from 10 of the 17 children in the intraobserver study were re-measured.

Both the systematic and random interobserver variation in the results of the remeasurements was consistent with the findings in the first measurements (Additional file 1). In the intraobserver remeasurements, we found that the coefficient of variation was reduced for all examined joints for both observers (data not shown).

## Discussion

To our knowledge, this is the first study that describes and quantifies inter- and intraobserver reliability of US assessments of cartilage thickness in large as well as small joints from clinically dominant joint regions in a large group of healthy children.

Prior to the implementation of US as a reliable method for detecting and monitoring the disease process in JIA patients, validity assessment is crucial. Our study contributes to the necessary description of the precision of this method (defined as how good a method reflects the closeness of agreement between different measurements of the same quantity). In addition we document that the recommended EULAR standard scan is applicable in a pediatric setting.

Our data showed a tendency to a somewhat small, but systematic interobserver variation, which was not related to joint size. This despite the use of standardized scanning settings and well-defined bony landmarks. The fact, that it is not possible for two observers to replicate the exact same location of the imaging plane could be part of the explanation. In accordance with Castriota-Scanderberg et al and our own previous study, small differences in transducer angulations can result in different measurements of the articular cartilage[12,13,19].

Fredberg an co-workers have documented that transversal scans increase the possibility of errors in measurement due to the fact that the transducer can be tilted in four planes (medially, laterally, proximally and distally) compared to only two planes in a longitudinal approach [18]. They measured Achilles and patellar tendons, while in our study this could very well explain part of the systematic interobserver variation for the knee measurements.

Also difficulties in replicating the exact same positioning of the patients joint from examination to examination could contribute to the systematic difference in the interobserver data.

This is consistent with the findings of Castriota-Scanderberg and colleagues who described the importance of standardizing the positioning[13] of the patients joints when studying inter- and intraobserver variation of cartilage thickness assessments in the hip and knee joint.

The random variation influence on the total measurement error seems to be mostly influenced by the "within joint" factor for both the inter- and intraobserver variation and there was no changes in this factors influence on the total measurement error after re-measuring.

The total variation between observers and the intraobserver variation was markedly larger when measuring the wrist compared to the other joints. As has also previously been stressed [2], this could be due to the complicated anatomical structure in this region and by the fact of investigating a growing joint. Differentiation between articular cartilage and immature growth cartilage is challenging leading to difficulties in establishing well-defined anatomical landmarks.

In a recent study we have tried to accede to this problem by comparing US wrist cartilage thickness to MRI measurements defined as "gold standard" [20]. We found, that the best approach for assessing wrist cartilage thickness was to measure according to the articular surface between the distal radial head and the scaphoid bone. Important when investigating JIA patients with often restricted limitation of motion interfering with the capability of optimal joint positioning for standardized US measurements[17].

In the finger joints, we found clear cartilage demarcations, especially in the MCP joints which is consistent with a relatively low inter- and intraobserver variability. Karmazyn and colleagues [21] used the same approach for evaluation of affected MCP joints but could not demonstrate that US was more significant in evaluating cartilage thinning. These results of Karmazyn and colleagues is most probably due to missing data regarding expected normal cartilage thickness in children of different ages, data which our group is in progress describing.

In the PIP joints, the significant difference in the interobserver study could be due to often blurred cartilage demarcation to the underlying cortex. Probably, this is due to the positioning of the probe which is critical in obtaining an interpretable image and a slight alteration in the angle of the probe in relation to the skin surface or a variation in the amount of gel used can distort the image obtained and increase the occurrence of artifacts.

In this study we found no difference in joint cartilage thickness between the left and right extremity in any of the examined joint of the healthy children. This observation is of particular interest when US measurements of cartilage thickness is implemented in a JIA patient group. Thus, the possibility of using the non-affected extremity as a reference along with an age- and sex-related reference value for cartilage thickness in healthy children could be used to determine the damage of the joint cartilage.

Further studies are needed to establish standard reference values for cartilage thickness in large and small joints according to age and gender in a healthy pediatric population. Such reference values would be important in a scoring system for routine US cartilage thickness measurements in JIA patients as a part of detecting and monitoring the disease process.

## Conclusion

US cartilage thickness measurements seems to have good inter- and intraobserver agreement in evaluation of cartilage thickness in synovial joints in children and that the recommended EULAR guidelines for standard scans of cartilage thickness is applicable in a paediatric setting.

We found that US was a well-accepted bed-side imaging tool, for evaluation of cartilage affection in synovial joints is children and very well could become the extended arm of the paediatric rheumatologist in the daily clinical examination.

## Competing interests

The authors declare that they have no competing interests.

## Authors' contributions

AHS Participated in the design and coordination of the study, carried out all the US examinations, participated in the statistical analysis and was responsible for the overall draft of the manuscript.

MPF Participated in the design of the study, carried out all the US examinations and participated in the draft of the manuscript.

NTA was responsible for the statistical analysis and participated in the draft of the manuscript.

ES Participated in the design of the study and drafting of the manuscript.

TH Participated in the design of the study and the draft of the manuscript.

## Supplementary Material

Additional file 1 **Table S1**. Interobserver variation of ultrasound cartilage thickness measurements in large and small joints in 74 healthy children. Click here for file

Additional file 2 **Table S2**. Intraobserver variation (observer I) in US measurements of articular cartilage thickness in 17 healthy children. Click here for file

Additional file 3 **Table S3**. Intraobserver variation (observer II) in US measurements of articular cartilage thickness in 17 healthy children. Click here for file

Additional file 4 **Table S4**. Cartilage thickness measurements for left and right extremity in the five examined joints in 74 healthy children – systemic and random variation. Click here for file
