# Supplementary material for: Inter -and intraobserver variation of ultrasonographic cartilage thickness assessments in small and large joints in healthy children
Source: Pediatr Rheumatol Online J. 2009 Jun 4;7:12. doi: 10.1186/1546-0096-7-12 (PMC2694801; doi:10.1186/1546-0096-7-12)
Supplement: Additional file 1 — Table S1. Interobserver variation of ultrasound cartilage thickness measurements in large and small joints in 74 healthy children. [file 1546-0096-7-12-S1.pdf]

Table 1 Interobserver variation of ultrasound cartilage thickness measurements in large and small joints in 74 healthy children

	**Systematic Variation[1]** **(between observer I and observer II)**		**Random Variation[2]**		**Total variation**
	Mean difference[3] in mm	*p*	SD* "within child" in mm	SD° "within joint" in mm	SD (CV[4]) in mm
**Knee**	0.26	<.0001	0.21	0.24	0.32 (9.5%)
**Ankle**	-0.14	<.0001	0.07	0.18	0.19 (19.9%)
**Wrist**	0.08	0.172	0.24	0.35	0.43 (26.9%)
**MCP**	-0.11	<.0001	0.06	0.10	0.12 (11.9%)
**PIP**	-0.09	<.0001	0.08	0.10	0.13 (18.2%)

[1]Systematic variation = whether one observer has a tendency to measure thicker or thinner cartilage thickness) [2]Random variation (e.g. biological variation within children) [3]Difference between cartilage thickness measurement observer I and observer II in mm. *SD=Standard deviation within child, refers to the variation in SD in mm between the right and left extremity °SD=Standard deviation within joint, refers to the SD in mm between observers within the same side extremity [4] Coefficient of variation is calculated from the relative differences of measurements (CV=SD/mean x 100)
