# Supplementary material for: Inter -and intraobserver variation of ultrasonographic cartilage thickness assessments in small and large joints in healthy children
Source: Pediatr Rheumatol Online J. 2009 Jun 4;7:12. doi: 10.1186/1546-0096-7-12 (PMC2694801; doi:10.1186/1546-0096-7-12)
Supplement: Additional file 3 — Table S3. Intraobserver variation (observer II) in US measurements of articular cartilage thickness in 17 healthy children. [file 1546-0096-7-12-S3.pdf]

Table 3. Intraobserver variation (observer II) in US measurements of articular cartilage thickness in 17 healthy children

Observer II

	Systematic Variation[1] (Day I and Day II)		Random Variation[2]		Total variation
	Mean difference[3] in mm	*p*	SD* "within child" in mm	SD° "within joint" in mm	SD (CV[4]) in mm
**Knee**	-0.21	0.053	0.23	0.24	0.34 (9.3%)
**Ankle**	-0.04	0.555	0.00	0.24	0.24 (20.9%)
**Wrist**	-0.03	0.751	0.21	0.29	0.36 (25.6%)
**MCP**	0.09	0.057	0.08	0.13	0.15 (11.9%)
**PIP**	0.05	0.297	0.09	0.13	0.16 (19.6%)

1: Two weeks between the first a second measurement of cartilage thickness. [2]Random variation (e.g. biological variation within children) [3]Difference between cartilage thickness measurement day I and day II in mm. *SD=Standard deviation within child, refers to the variation in SD in mm between the right and left extremity °SD=Standard deviation within joint, refers to the SD in mm between observers within the same side extremity [4] Coefficient of variation is calculated from the relative differences of measurements (CV=SD/mean x 100)
