# Supplementary material for: Inter -and intraobserver variation of ultrasonographic cartilage thickness assessments in small and large joints in healthy children
Source: Pediatr Rheumatol Online J. 2009 Jun 4;7:12. doi: 10.1186/1546-0096-7-12 (PMC2694801; doi:10.1186/1546-0096-7-12)
Supplement: Additional file 4 — Table S4. Cartilage thickness measurements for left and right extremity in the five examined joints in 74 healthy children – systemic and random variation. [file 1546-0096-7-12-S4.pdf]

Table .4 Cartilage thickness measurements for left and right extremity in the five examined joints in 74 healthy children – systematic and random variation

	**Systematic variation** (between left and right extremities) *****		**Random Variation ***
	Cartilage Thickness Mean difference in mm°	*p*	SD in mm
**Knee**	0.029	0.333	0.06
**Ankle**	0.020	0.348	0.00
**Wrist**	-0.013	0.756	0.00
**MCP**	-0.014	0.347	0.00
**PIP**	0.011	0.389	0.10

°Mean difference in mm between left and right extremities SD= standard deviation
